# Deletion of Rv2571c Confers Resistance to Arylamide Compounds in Mycobacterium tuberculosis

**DOI:** 10.1128/AAC.02334-20

**Published:** 2021-04-19

**Authors:** Catherine D. Shelton, Matthew B. McNeil, Julie V. Early, Thomas R. Ioerger, Tanya Parish

**Affiliations:** a Infectious Disease Research Institute, Seattle, Washington, USA; b Department of Computer Science and Engineering, Texas A&M University, College Station, Texas, USA; c Center for Global Infectious Disease Research, Seattle Children’s Research Institute, Seattle, Washington, USA

**Keywords:** antibacterial, antitubercular, antibiotic resistance, drug uptake

## Abstract

Tuberculosis, caused by Mycobacterium tuberculosis, is an urgent global health problem requiring new drugs, new drug targets, and an increased understanding of antibiotic resistance. We have determined the mode of resistance to be a series of arylamide compounds in M. tuberculosis. We isolated M. tuberculosis resistant mutants to two arylamide compounds which are inhibitory to growth under host-relevant conditions (butyrate as a sole carbon source). Thirteen mutants were characterized, and all had mutations in Rv2571c; mutations included a premature stop codon and frameshifts as well as nonsynonymous polymorphisms. We isolated a further 10 strains with mutations in Rv2571c with resistance. Complementation with a wild-type copy of Rv2571c restored arylamide sensitivity. Overexpression of Rv2571c was toxic in both wild-type and mutant backgrounds. We constructed M. tuberculosis strains with an unmarked deletion of the entire Rv2571c gene by homologous recombination and confirmed that these were resistant to the arylamide series. Rv2571c is a member of the aromatic amino acid transport family and has a fusaric acid resistance domain which is associated with compound transport. Since loss or inactivation of Rv2571c leads to resistance, we propose that Rv2571c is involved in the import of arylamide compounds.

## Abstract (precis)

Tuberculosis, caused by Mycobacterium tuberculosis, is an urgent global health problem requiring new drugs, new drug targets, and an increased understanding of antibiotic resistance. We have determined the mode of resistance to be a series of arylamide compounds in M. tuberculosis.

## INTRODUCTION

Tuberculosis remains a major global health problem with ∼10 million new cases and over 1 million deaths annually (1). The Covid-19 pandemic is likely to exacerbate the situation with fewer cases being detected and treated (1). Antibiotic resistance is a major problem with multidrug-resistant and extremely drug-resistant strains in circulation. There is an urgent need for new drug targets as well as an increased understanding of antibiotic resistance in the causative pathogen Mycobacterium tuberculosis (2, 3).

In the last decade, numerous high-throughput screening campaigns have been conducted in order to identify a new chemical matter that could be developed as novel antitubercular agents (4). We (and others) have conducted several whole-cell phenotypic screens under different conditions to identify chemical matter with different biological activities (4). In particular, we have used conditions which reflect aspects of the *in vivo* infection environment (4). Since M. tuberculosis utilizes fatty acids as a carbon source *in vivo*, we previously developed a whole-cell screen with butyrate as the sole carbon source (5). Using this high-throughput screen, we identified a series of aryl amides with good activity against M. tuberculosis. These compounds are of interest because they are only active against M. tuberculosis using butyrate as the sole carbon source; in the presence of glucose as the sole carbon source, compounds lose activity. Since the activity is only seen under particular culture conditions, it is unlikely that the compounds target a promiscuous target such as MmpL3 or QcrB, which are involved in cell wall biosynthesis and the electron transport chain, respectively (6–8).

We have previously conducted extensive structure-activity relationship and structure-property relationship studies in order to ascertain whether this series was developable (unpublished). In parallel, we were interested in determining the target and mechanism of resistance to the series of compounds. In this study, we demonstrate that resistance is due to a loss of activity of the putative transport system Rv2571c.

## RESULTS

**FIG 1:**
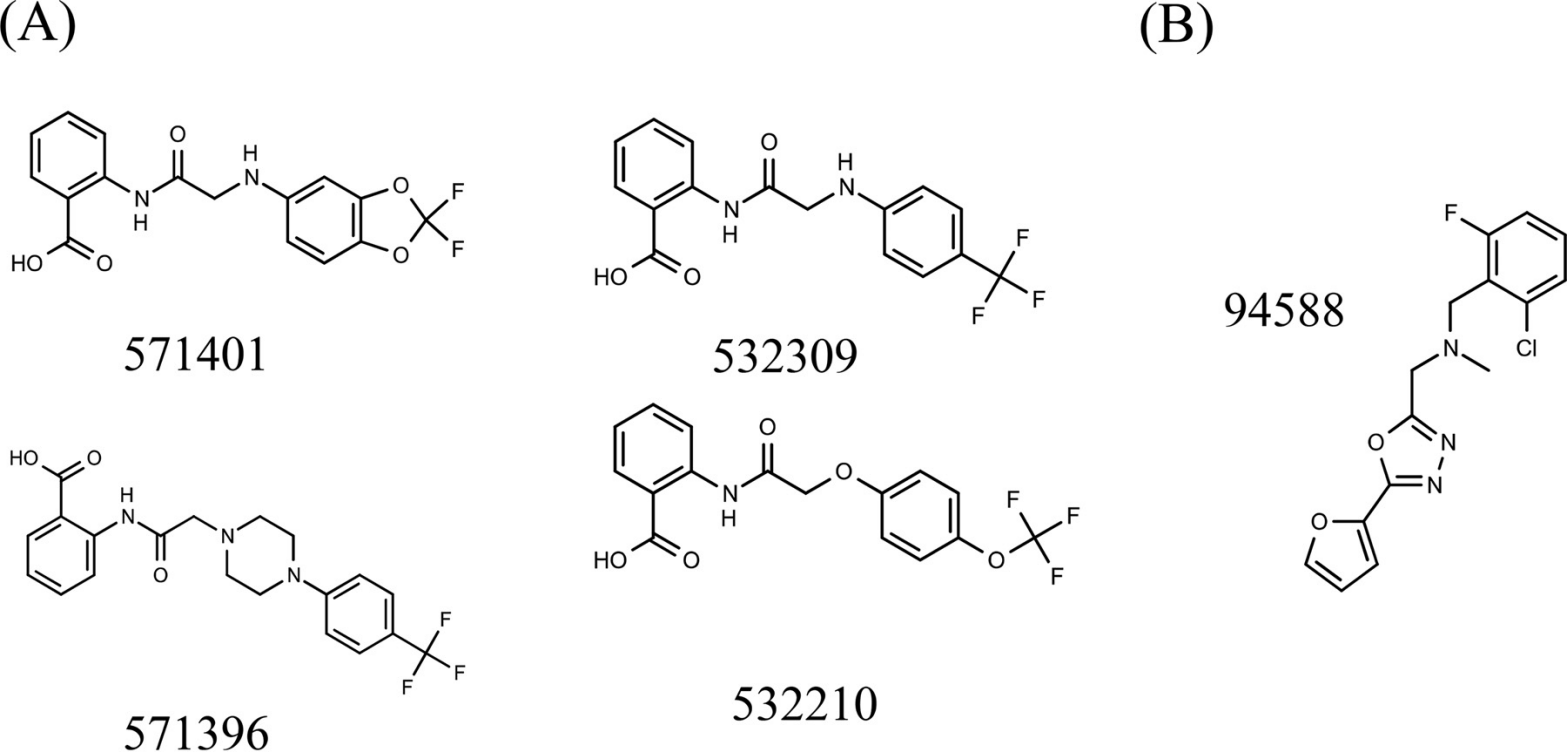
Compounds selected for this study. (A) Arylamide (AMI) series. (B) Aryl oxadiazole (ARO) series (9).

We identified the arylamide (AMI) series of compounds from a phenotypic screen against M. tuberculosis cultured with butyrate as the sole carbon source (9). Compounds were inhibitory to growth only when M. tuberculosis was cultured using butyrate but had no activity in cultures utilizing glucose as the sole carbon source (unpublished data). We were interested to determine mechanism(s) of resistance to this series and/or to determine the intracellular target(s). We selected representative compounds with good activity against wild-type M. tuberculosis (Fig. 1 and Table 1).

**TABLE 1:** Activity of compounds used in this study^*a*^

Compound	Series	MIC_90_ (μM)	MIC_99_ (μM)
571401	AMI	0.87 ± 0.05	0.40
571396	AMI	1.0 ± 0.1	0.80
532310	AMI	0.79 ± 0.04	0.20
532309	AMI	0.65 ± 0.3	0.40
94588	ARO	0.79 ± 0.5	ND

a MIC_90_ was measured in liquid medium and MIC_99_ was measured on solid medium (N = 2) containing butyrate as the sole carbon source (5). AMI, arylamide series. ARO, aryl oxadiazoles (9). ND, no data.

### Resistant isolates have mutations in Rv2571c.

We previously used the approach of isolating resistant mutants in combination with whole-genome sequencing to identify the bacterial targets of novel compounds (10). We used the same approach to identify spontaneous resistant mutants for the AMI compound series. Since compounds were only active if butyrate was the sole carbon source, we used agar containing butyrate as the sole carbon source to isolate resistant mutants. We isolated resistant mutants to two representative compounds from the series (532309 and 571401) on plates containing 5× MIC_99_ (solid agar). The frequency of resistance was ∼1 × 10^−8^ for both compounds. We confirmed resistance by streaking colonies onto plates containing 5× MIC_99_. We confirmed 13 strains with resistance to the parent compounds. We further confirmed that strains were resistant to the class of compounds by determining the MIC_90_ in liquid medium (Table 2). All strains showed high resistance to the AMI compounds but were not resistant to another class of compounds with butyrate-specific activity (the aryl oxadiazole “ARO” series) (5).

**TABLE 2:** M. tuberculosis strains with resistance to arylamide compounds^*a*^

Strain	AMI (MIC_90_ μM)				ARO (MIC_90_ μM)
	571401	571396	532310	532309	94588
Wild-type	0.87	1.0	0.79	0.65	0.79
LP-0571401-RM1	>20	>20	>20	>20	3.5
LP-0571401-RM2	>20	>20	>20	>20	3.6
LP-0571401-RM3	>20	>20	>20	>20	1.6
LP-0571401-RM4	18	>20	15	9.4	2.0
LP-0571401-RM5	>20	>20	>20	>20	2.9
LP-0571401-RM6	>20	>20	>20	>20	5.5
LP-0571401-RM7	>20	>20	>20	>20	3.8
LP-0532309-RM1	>20	>20	>20	>20	2.8
LP-0532309-RM2	>20	>20	>20	>20	4.6
LP-0532309-RM3	>20	>20	>20	>20	3.1
LP-0532309-RM4	>20	>20	>20	>20	1.7
LP-0532309-RM5	>20	>20	>20	>20	2.2
LP-0532309-RM7	>20	>20	>20	16	2.0

a Resistant mutants were isolated on plates containing 5× MIC_99_ for compounds 571401 and 532309. Resistance was confirmed by streaking onto plates containing 5× MIC_99_. MIC_90_ was measured in liquid medium (5). AMI, arylamide series. ARO, aryl oxadiazoles.

We sequenced the entire genome for two strains (LP-0571401-RM1 and LP-0571401-RM2) and identified genetic variations, including single nucleotide polymorphisms and insertion/deletions. There was one mutation in common between the two strains in MutT1. Mutations were noted in four other genes. Strain LP-0571401-RM1 had MutT1 E252D, EmbB S651R, and Rv2571c A204fs; strain LP-0571401-RM2 had Rv0047c R143C, Rv2011c A118S, and MutT1 E2525D. In order to determine which mutations were associated with resistance, we sequenced all five genes in the 13 resistant isolates (Table 3). All of the strains showed mutations in Rv2571c, although from the sequencing chromatograms it appeared that two of the isolates were not pure. Eleven of the strains were pure isolates with Rv2571c single nucleotide polymorphisms (SNPs). Several of the strains had mutations in Rv2571c alone. We found an additional SNP in Rv2571c for one of the strains which had been fully sequenced, which is not unusual if coverage is low or data calls are ambiguous.

**TABLE 3:** Sequence analysis of resistant isolates^*a*^

Strain	MutT1 (Rv2985)	EmbB (Rv3795)	Rv2011c	Rv0047c	Rv2571c
LP-0532309-RM1	None	None	None	None	A311D
LP-0532309-RM2	None	None	None	None	A311D
LP-0532309-RM3	None	None	None	None	R149H
LP-0532309-RM4	None	S651R	None	None	R149H
LP-0532309-RM5	E252D	S651R	None	None	A160P^*b*^
LP-0532309-RM7	None	S651R	None	None	Q29R^*b*^
LP-0571401-RM1	E252D	S651R	None	None	A204fs
LP-0571401-RM2	E252D	None	A118S	R143C	V243fs
LP-0571401-RM3	None	S651R	None	None	D152G
LP-0571401-RM4	E252D	None	None	None	Y256^*b*^
LP-0571401-RM5	None	None	None	None	V119A
LP-0571401-RM6	None	None	None	None	A140P
LP-0571401-RM7	None	None	None	None	L159Q

a Genes were sequenced in each strain. fs, frameshift.

b The chromatogram suggested a mixed population of mutant and wild-type alleles.

We isolated additional resistant mutants on solid plates using compound 571401 and confirmed resistance by growth on plates containing 5× MIC_99_. We sequenced Rv2571c in 10 resistant isolates, all of which had novel spontaneous mutations in Rv2571c (Table 4). Since all of the mutants had SNPs in Rv2571c, it supported our hypothesis that Rv2571c plays a role in resistance to AMI compounds. Several of the mutations in Rv2571c were frameshifts or premature stop codons, suggesting that inactivation of the gene conferred resistance. Other mutations included those that are predicted to affect protein function; for example, mutations to a proline would be expected to alter protein structure. Some mutations, e.g., V119A and R149H, were minor but could still have an effect on protein structure depending on location.

**TABLE 4:** Additional mutations in Rv2571c that confer resistance to arylamides

Strain	Rv2571 mutation
1	D43^*a*^
2	A140P
3	D43^*a*^
4	V243C
5	D43^*a*^
6	L81P
7	R149L
8	V119A
9	D43^*a*^
10	Y256^*a*^

a The chromatogram suggested a mixed population of mutant and wild-type alleles.

**FIG 2:**
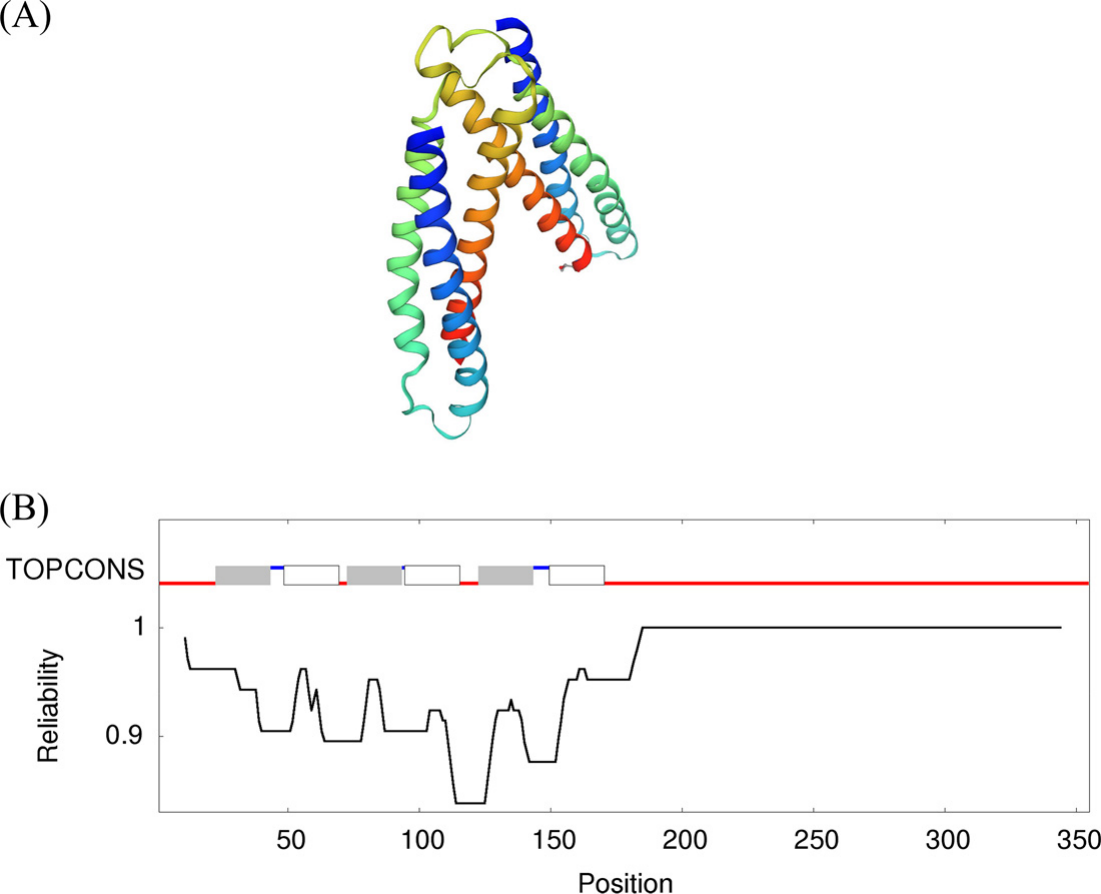
Analysis of Rv2571c. (A) Homology model of Rv2571c residues 17 to 271 (P9WL89) based on Pseudomonas aeruginosa PhoU (SMTL ID: 4q 25.1). (B) Transmembrane prediction using TOPCONS (13).

There is no protein structure available for Rv2571c or any related proteins. A partial model covering amino acids 17 to 271 (P9WL89) based on the structure of the 100 amino acids of Pseudomonas aeruginosa PhoU (SMTL ID: 4q 25.1) is available (11, 12); Rv2571c has 14.5% identity with PhoU (Fig. 2). The protein has six transmembrane helices followed by an intracellular domain as predicted by TOPCONS (13). The mutations we found mapped throughout the protein in the transmembrane helices (TM1: Q29, D43; TM3: L81P; TM4/5 junction: V199A; TM5: A140P; TM6: R149, D152, A160) and the intracellular domain (A204, V243, Y256, A311).

### Restoration of Rv2571c function leads to AMI sensitivity.

In order to determine if Rv2571c inactivation was responsible for resistance, we complemented one of the resistant strains (LP-0532309-RM1 carrying Rv2571c_A311D_ allele) with a functional copy using an extrachromosomal plasmid in which expression of the gene was under the control of a tetracycline-inducible promoter (pOE:Rv2571c) (14, 15). Introduction of the plasmid was sufficient to restore sensitivity to AMI compounds, confirming that resistance was due to a lack of Rv2571c activity (Table 5). This occurred even in the absence of anhydrotetracycline (ATc), since this system has leaky expression (14, 15). We were not able to determine MICs in the presence of ATc due to toxicity (see below). Introduction of the Rv2571c expression plasmid into the wild-type background had no effect on MIC (data not shown).

**TABLE 5:** Complementation with functional Rv2571c restores sensitivity to arylamides^*a*^

Strain	MIC (μM)			
	**571401**	**571396**	**532310**	**532309**
LP-0532309-RM1	>200	>200	>200	>200
LP-0532309-RM1 (pOE:Rv2571)	0.55 ± 0.6	1.6 ± 0.07	1.2 ± 0.7	nt

*^a^*MIC_90_ for AMI compounds was determined in liquid medium containing butyrate as the sole carbon source. LP-0532309 carries Rv2571c_A311D_. pOE:Rv2571c is a plasmid carrying wild-type Rv2571c under the control of an ATc-inducible promoter. Data are average ± standard deviation for two independent runs. nt, not tested.

**FIG 3:**
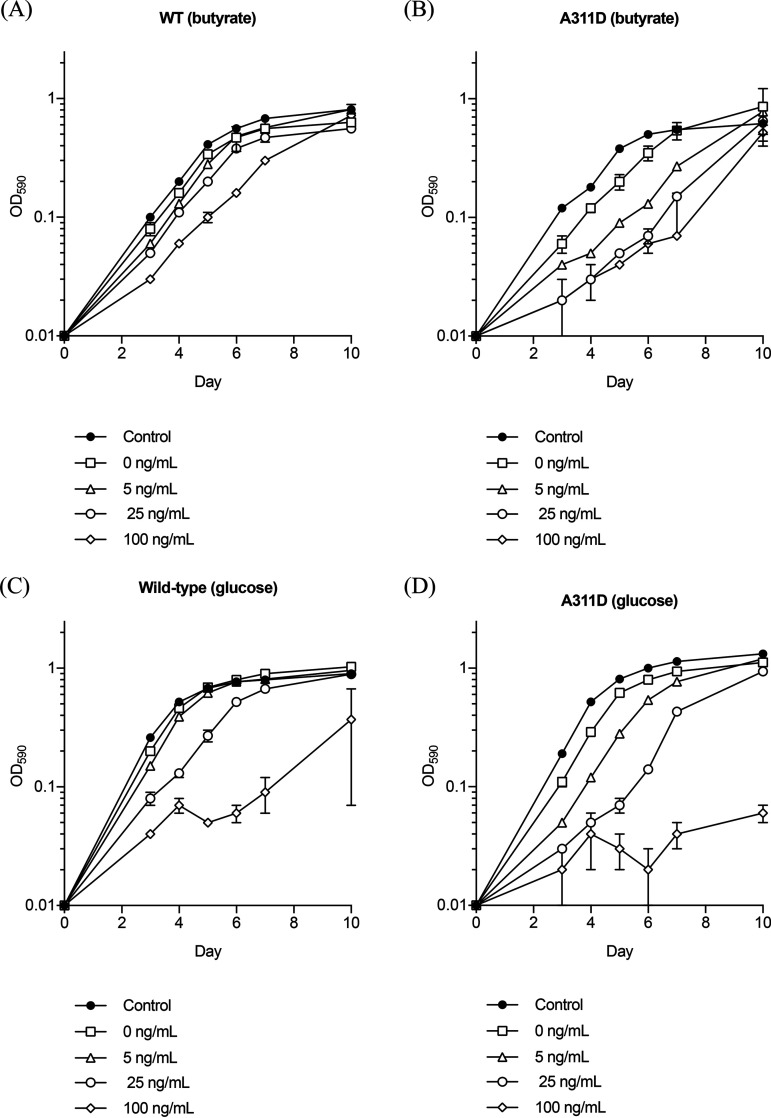
Overexpression of Rv2571C is toxic. M. tuberculosis strains carrying an ATc-inducible, Rv2571c expression plasmid (pOE:Rv2571c) were grown using either butyrate or glucose as the sole carbon source. The control was empty plasmid. Growth was monitored by OD_590_ in increasing concentrations of ATc (ng/ml). Data are the mean ± standard deviation of three independent cultures. (A and B) Butyrate as the carbon source. (C and D) Glucose as the carbon source. WT, wild-type strain Rv2571c_wt_; A311D, strain LP-0532309-RM1 Rv2571c_A311D_.

Since we noted low growth in the strains expressing Rv2571c in the presence of ATc, we determined whether overexpression was toxic. We looked at the effect of overexpression of Rv2571c on the growth rate of the wild-type and resistant mutant strains (Fig. 3). We induced expression of Rv2571c using the ATc-inducible system, allowing us to titrate expression (higher expression as ATc is increased). When butyrate was used as the sole carbon source, there was a decrease in growth rate in both the wild-type and the knockout strains even in the absence of ATc. The effect became more pronounced with higher expression levels, demonstrating significant growth inhibition at the highest concentrations of ATc (Fig. 3A and B). When glucose was used as a sole carbon source, there was also a reduction in growth rate. Again, the effect was ATc concentration-dependent with both wild-type and knockout strains showing a marked reduction in growth at 100 ng/ml ATc (Fig. 3C and D).

### Deletion of Rv2571c confers resistance to AMI compounds.

**FIG 4:**
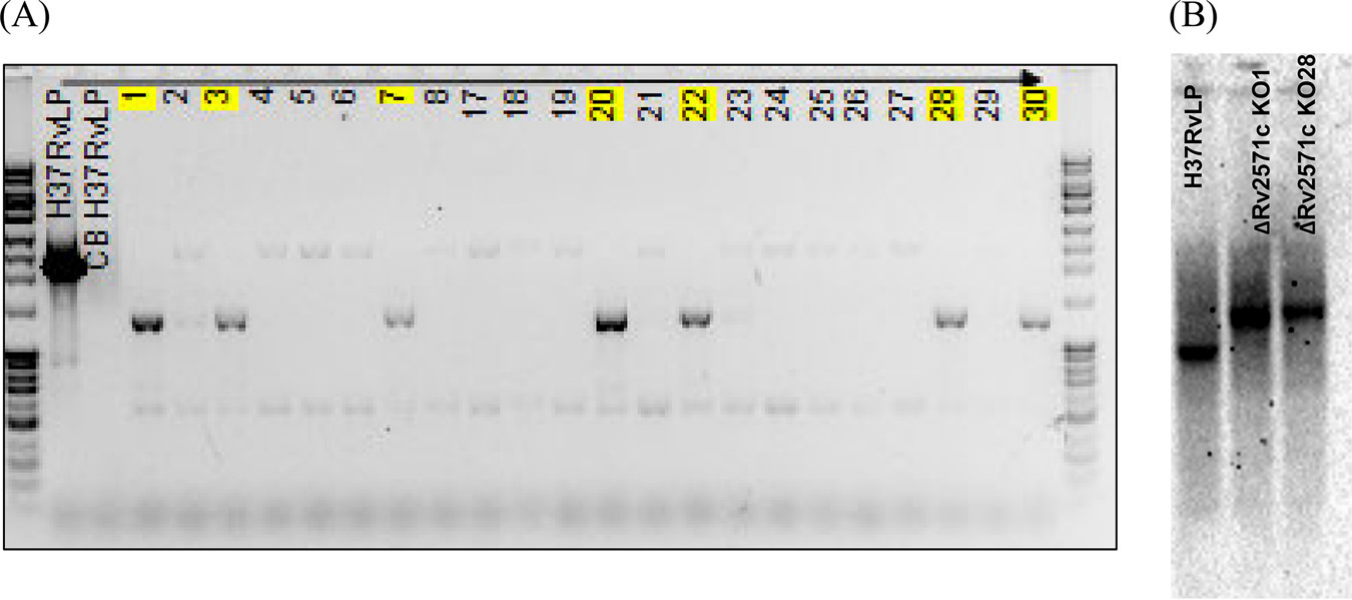
Selection and confirmation of Rv2571c deletion strains. (A) PCR and (B) Southern blotting were used to confirm the presence of the wild-type or deletion strains in DCOs. (A) PCR amplification from confirmed DCOs. H37RvLP, wild-type strain. Numbers, double crossover strains. Yellow highlights indicate deletion strains. L, 1 kb ladder (Promega). (B) Southern analysis for two deletion strains. DNA was digested with NcoI and the hybridization probe was the entire gene. Expected sizes were (A) wild-type 2.5 kb and deletion 1.3 kb and (B) wild-type 3.5 kb and deletion 6.7 kb.

**FIG 5:**
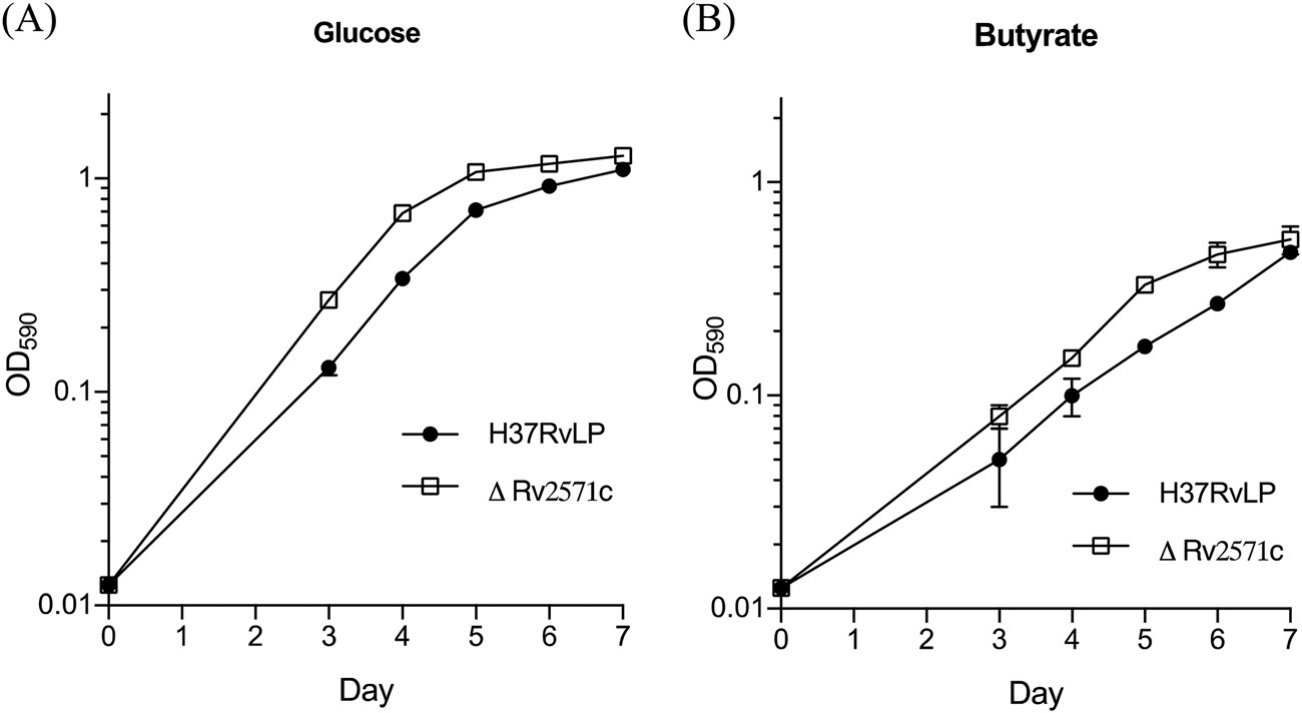
Rv2571c is not essential for growth in butyrate. M. tuberculosis strains were grown using either (A) butyrate or (B) glucose as the sole carbon source. Data are the mean ± standard deviation of three independent cultures.

In order to confirm that Rv2571c inactivation leads to resistance to AMI compounds, we constructed an unmarked in-frame deletion mutant strain by homologous recombination (16). We isolated double crossover (DCO) strains containing either wild-type or deletion allele. Strains were screened by PCR and confirmed by Southern analysis (Fig. 4). We determined whether Rv2571c was essential for growth using butyrate as the sole carbon source (Fig. 5); the deletion strain showed no reduction in growth rate using either glucose or butyrate as the sole carbon source. This confirms that Rv2571c is not essential for growth utilizing butyrate.

We tested the deletion strain for resistance to the compound by streaking onto agar plates containing 5× MIC_99_ compound and obtained growth. We further confirmed resistance by testing in liquid medium (Table 6). Two strains carrying a deletion allele (KO1 and KO7) were resistant, whereas a DCO carrying the wild-type copy (KO18) was as sensitive as the wild-type parental strain. These data confirm that Rv2571c is required for sensitivity to AMI compounds and that its absence leads to resistance.

**TABLE 6:** Deletion of Rv2571 leads to resistance to arylamide compounds^*a*^

		MIC_90_ (μM)			
**Strain**	**Rv2571 allele**	**571401**	**571396**	**532310**	**532309**
**H37RvLP**	Wild-type	1.2	2.1	0.9	0.6
**DCO1**	Deletion	>20	>20	>20	>20
**DCO7**	Deletion	>20	>20	>20	>20
**DCO18**	Wild-type	1.2	1.4	0.6	0.6

a MIC_90_ was determined in liquid medium containing butyrate as the sole carbon source.

## DISCUSSION

We have demonstrated that inactivation of Rv2571c leads to resistance to arylamide compounds. Rv2571c is not essential under conditions in which the compounds are active, suggesting that inhibition leading to loss of function is not the mode of action of this compound series.

A second alternative is that Rv2571c encodes an uptake system required for import of the compound into the cell, since deletion of such a system would result in resistance. Rv2571c is predicted to encode a transmembrane protein of the aromatic amino acid exporter family and contains a FUSC2 (fusaric acid resistance protein-like) domain (E value of 1.1e^−13^) (17). These proteins are involved in export of fusaric acid (18); thus, it seems likely that Rv2571c is a membrane transporter protein. Since resistance was confirmed by deletion of Rv2571c, we hypothesize that this protein is involved in the import of compounds rather than efflux. In this scenario, mutations or deletion could lead to reduced uptake of the compound and lack of access to the intracellular target. In this scenario, the selectivity of the compounds would result from the conditional essentiality of the target; i.e., the target is only essential when the bacteria are grown using butyrate as the sole carbon source.

Of interest, another membrane protein, MmpL3, has also been reported as a compound importer in M. tuberculosis, and in this case, mutations also lead to resistance to the tetrahydropyrazo-pyrimidine-carboxamide compounds. However, since MmpL3 is an essential protein, deletion strains were not available for confirmation, but the true target was identified as EchA6 by pulldown assays. Future work to identify the target could include a number of approaches, including pulldown assays, use of an overexpression library or transcriptome, and metabolome studies (19).

The apparent toxicity during increased expression of Rv2571c could result from protein aggregation or as a general stress response. We think it more likely that it reflects the fact that the protein is normally expressed at a low level. Indeed, Rv2571c is under the negative control of the two-component regulatory system TcrA (14) and is one of the least abundant proteins in the cell, with only ∼30 copies/cell in exponentially growing bacteria (glucose as the carbon source) (20). Alternatively, it could suggest that the mutant allele still has residual function.

In conclusion, we have demonstrated that Rv2571c is required for the activity of the arylamide series against M. tuberculosis using butyrate as a sole carbon source. We hypothesize that Rv2571c is required for uptake of these compounds.

## MATERIALS AND METHODS

### Culture of M. tuberculosis.

M. tuberculosis H37Rv-LP (ATCC 25618) was cultured in Middlebrook 7H9 medium containing 10% vol/vol OADC (oleic acid, albumen, dextrose, catalase) supplement and 0.05% wt/vol Tween (7H9-Tw-OADC) or Middlebrook 7H9 medium supplemented with 5 g/liter bovine serum albumin (BSA) fraction V, 0.8 g/liter NaCl, 0.05% vol/vol Tyloxapol, and sodium butyrate (7H9-Ty-BT) at 5 mM (7H9-Ty-5BT) or 10 mM (7H9-Ty-10BT). Agar plates were prepared with Middlebrook 7H10 base supplemented with 5 g/liter BSA fraction V, 0.8 g/liter NaCl, and 5 mM butyrate. Where required, kanamycin was added at 20 μg/ml, hygromycin was added at 100 μg/ml, sucrose was added at 2% wt/vol, and anhydrotetracycline (ATc) was added at 5 to 100 μg/ml.

### Determination of MICs.

MICs in liquid medium were determined as described (5, 21). MIC_90_ was defined as the concentration required to inhibit growth by 90%. MIC_99_ on solid medium was determined as the lowest concentration which prevented the growth of 99% of colonies.

### Isolation of resistant mutants.

We isolated resistant mutants as previously described (10). Resistant mutants were isolated on plates containing 5× MIC_99_ for compounds 571401 and 532309. Whole-genome sequencing was performed as described (22). Mutations were confirmed by sequencing specific genes.

### Construction and complementation of deletion strains.

We constructed an unmarked deletion strain of Rv2571c as previously described (16). Briefly, a suicide vector containing ∼1 kb of sequence upstream and downstream of the gene was constructed (unmarked deletion) carrying hyg, kan, sacB, and *lacZ* genes. The upstream region was amplified using primers TCAGCAACGTAAGGAGT and TACCGCGACGAGGACTT; the downstream region was amplified using primers GCCGAGATCGAGGTT and GGATCCAGGTAGCCCGACACATA. Single crossover strains were generated by electroporation; double crossover strains were selected/screened on sucrose and X-Gal (5-bromo-4-chloro-3-indolyl-β-d-galactopyranoside) agar plates and confirmed by PCR amplification of the gene using primers AAACCGGAATGGGAGGAC and GTTGCTGAGCGGTAATGG. Deletion strains were confirmed by Southern blotting and sequencing. The deletion strain carried a precise deletion of the entire gene (start to stop codon). The complementing vector was generated by cloning a PCR product generated with primers TTAATTAAATAGATGCCCAGCCCA and TTAATTAATGGCCGAGATCGAGGTT into pDTCF or pDTNF (14).
